# The effect of convolving word length, word frequency, function word predictability and first pass reading time in the analysis of a fixation-related fMRI dataset

**DOI:** 10.1016/j.dib.2019.104171

**Published:** 2019-07-23

**Authors:** Benjamin T. Carter, Steven G. Luke

**Affiliations:** Brigham Young University, USA

**Keywords:** fMRI, Eye-tracking, Predictability, Reading

## Abstract

The data presented in this document was created to explore the effect of including or excluding word length, word frequency, the lexical predictability of function words and first pass reading time (or the duration of the first fixation on a word) as either baseline regressors or duration modulators on the final analysis for a fixation-related fMRI investigation of linguistic processing. The effect of these regressors was a central question raised during the review of *Linguistic networks associated with lexical, semantic and syntactic predictability in reading: A fixation-related fMRI study* [1]. Three datasets were created and compared to the original dataset to determine their effect. The first examines the effect of adding word length and word frequency as baseline regressors. The second examines the effect of removing first pass reading time as a duration modulator. The third examines the inclusion of function word predictability into the baseline hemodynamic response function. Statistical maps were created for each dataset and compared to the primary dataset (published in [1]) across the linguistic conditions of the initial dataset (lexical predictability, semantic predictability or syntax predictability).

**Table:** Specifications Table

Subject area	*Neuroscience, fMRI, Reading, Eye tracking*
More specific subject area	*Prediction of linguistic features during reading tasks*
Type of data	*Tables and figures*
How data was acquired	*Siemens 3T Tim Trio with a 12-channel receive only head coil (fMRI)* *SR Research Eyelink 1000 plus long-range MRI eye-tracker (eye tracking)* *Cambridge Systems MRI-safe LCD (stimulus presentation)*
Data format	*Analyzed*
Experimental factors	*No pretreatment was administered. Participants were required to be right-handed, native English speakers and literate, with normal 20/20 vision. Participants were recruited from the student body of Brigham Young University.*
Experimental features	*41 participants read 54 paragraphs and underwent concurrent eye tracking and fMRI.*
Data source location	*Provo, Utah, United States of America*
Data accessibility	*Analysis scripts and sample data can be found at:*https://github.com/btcarter/LinguisticPrediction *Full dataset can be found at: osf.io/7csxr*
Related Research Article	*Carter, B. T., Foster, B., Muncy, N., & Luke, S. G. (2019). Linguistic networks associated with lexical, semantic and syntactic predictability in reading: A fixation-related fMRI study. NeuroImage.*

**Table:** 

**Value of the data** • Fixation-related fMRI is a technique that combines eye-tracking and fMRI. In this technique, individual fixations are treated as events, and BOLD activation related to these fixation events is analyzed [2], [3], [4], [5]. The analysis includes parametric regressors associated with the currently fixated stimulus. • This technique has greater ecological validity than traditional approaches, especially when applied to reading. At the same time, it presents analytical challenges, requiring well designed and tightly fitted hemodynamic response functions. • Data can be used to determine which linguistic- and fixation-based regressors should be included in the baseline hemodynamic response function when using fixation-related fMRI to investigate reading.

## 1 Data

**Fig. 1:**
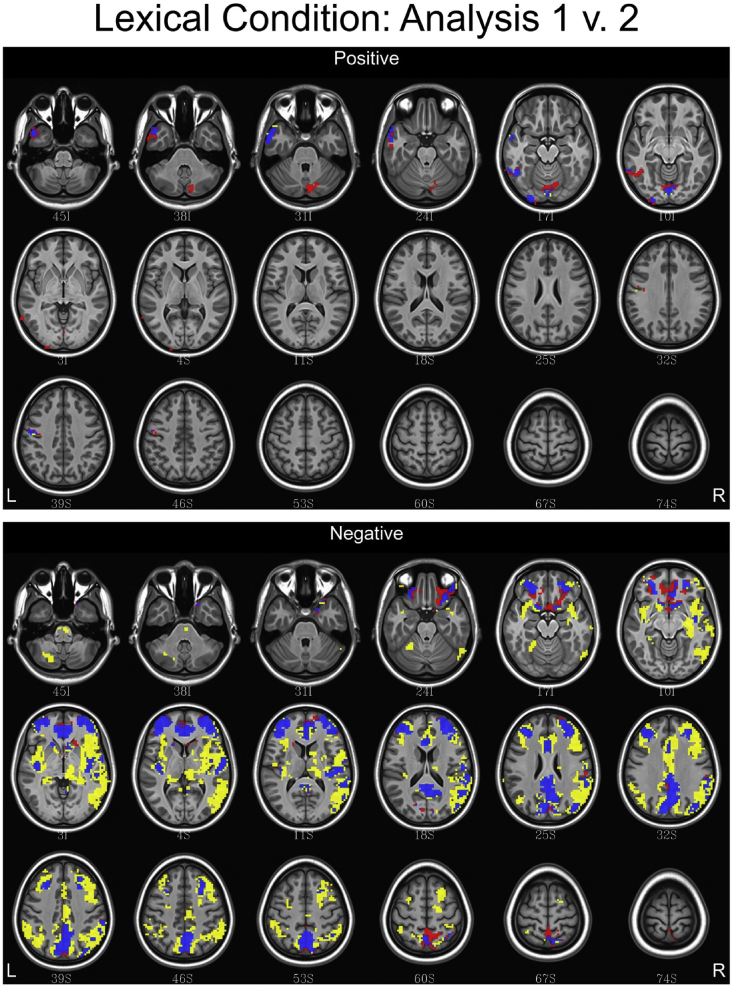
Conjunction maps comparing the location of significant voxels positively or negatively associated with Analysis 1 and Analysis 2 for the lexical condition. Voxels associated with Analysis 1 are red. Voxels associated with Analysis 2 are yellow. Voxels common to both analyses are blue.

**Fig. 2:**
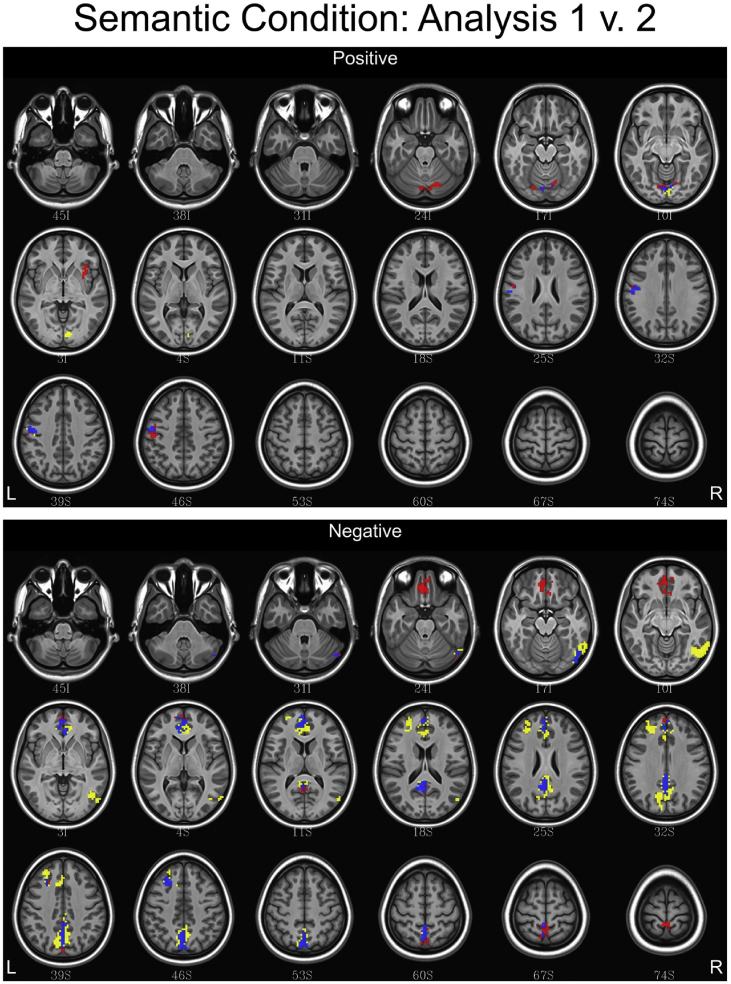
Conjunction maps comparing the location of significant voxels positively or negatively associated with Analysis 1 and Analysis 2 for the semantic condition. Voxels associated with Analysis 1 are red. Voxels associated with Analysis 2 are yellow. Voxels common to both analyses are blue.

**Fig. 3:**
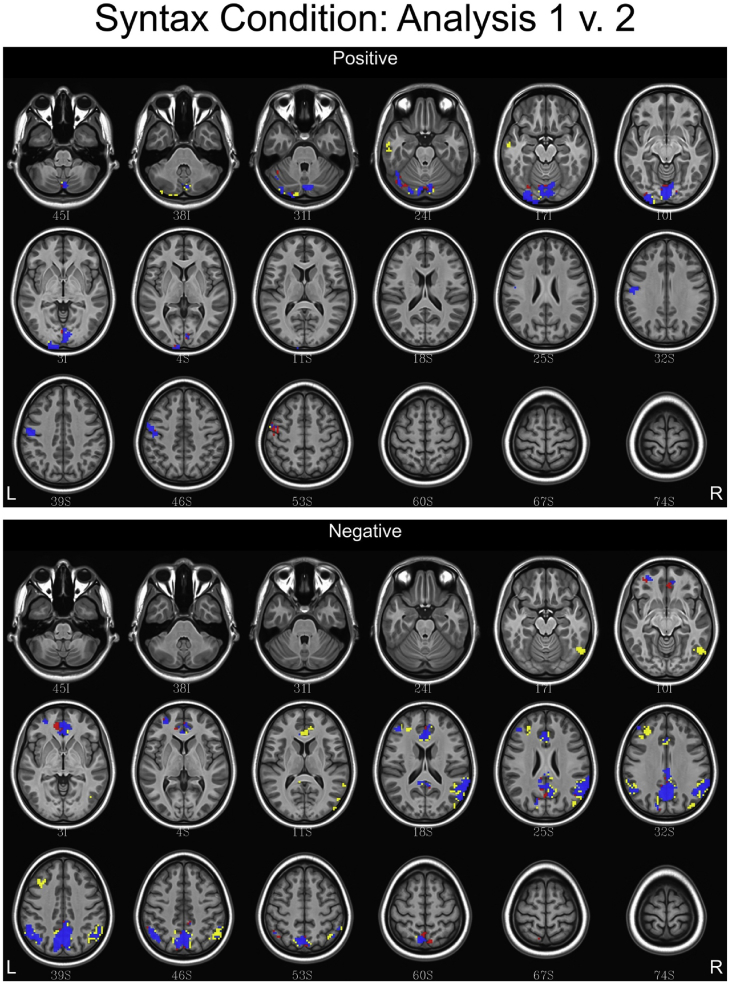
Conjunction maps comparing the location of significant voxels positively or negatively associated with Analysis 1 and Analysis 2 for the syntax condition. Voxels associated with Analysis 1 are red. Voxels associated with Analysis 2 are yellow. Voxels common to both analyses are blue.

**Fig. 4:**
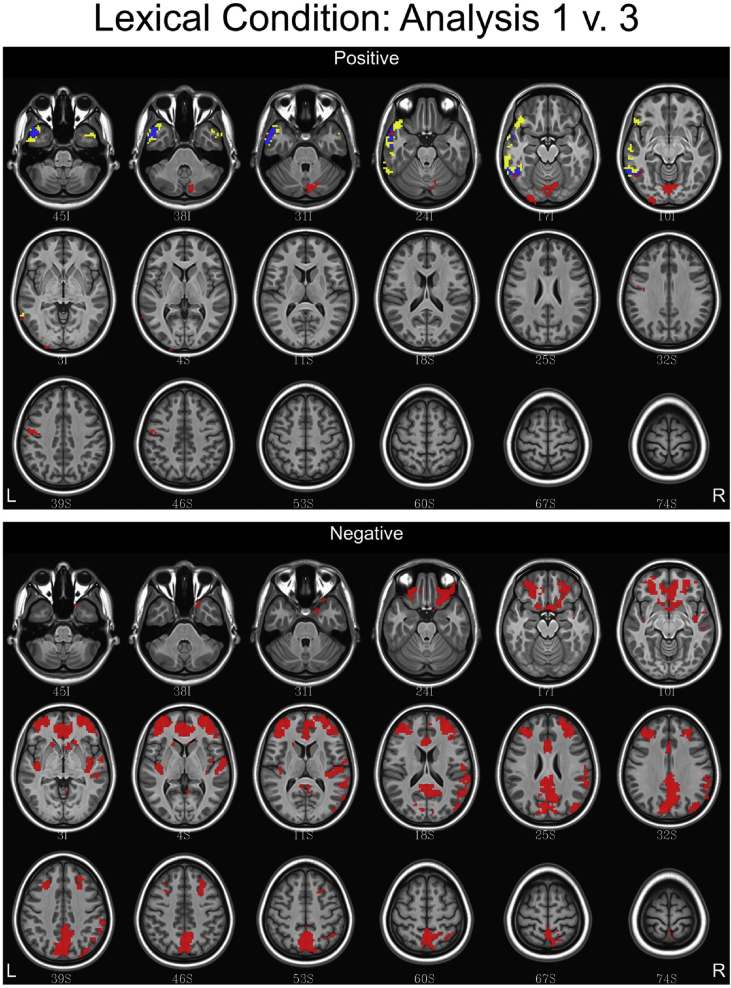
Conjunction maps showing the location of significant voxels positively or negatively associated with Analysis 1 and Analysis 3 for the lexical condition. Voxels associated with Analysis 1 are red. Voxels associated with Analysis 3 are yellow. Voxels common to both analyses are blue.

**Fig. 5:**
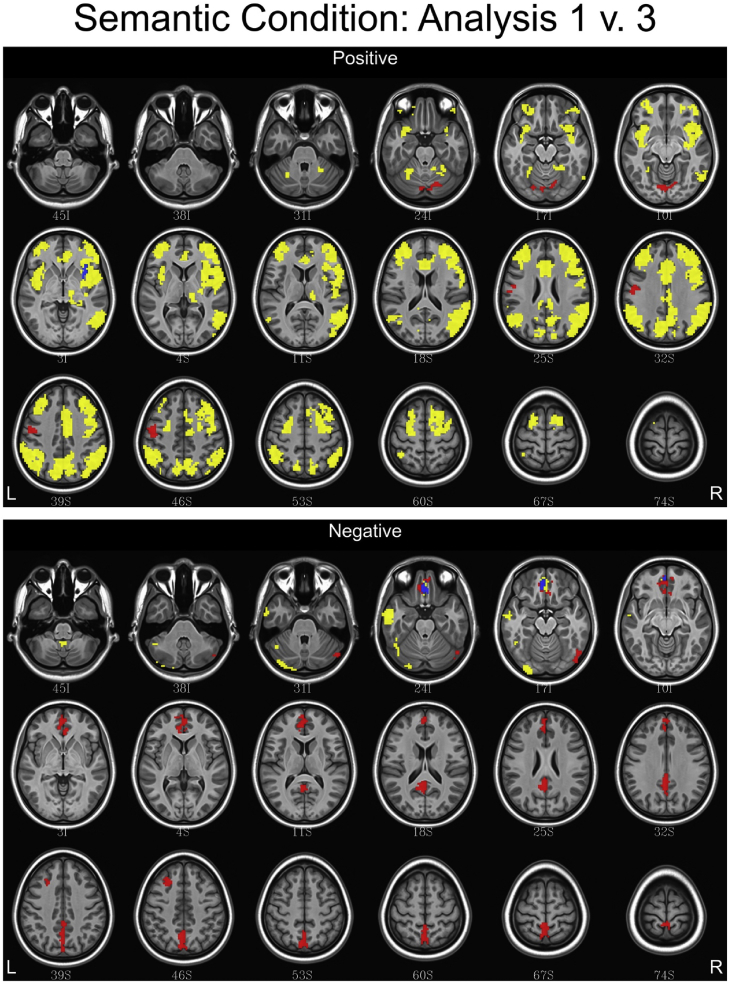
Conjunction maps showing the location of significant voxels positively or negatively associated with Analysis 1 and Analysis 3 for the semantic condition. Voxels associated with Analysis 1 are red. Voxels associated with Analysis 3 are yellow. Voxels common to both analyses are blue.

**Fig. 6:**
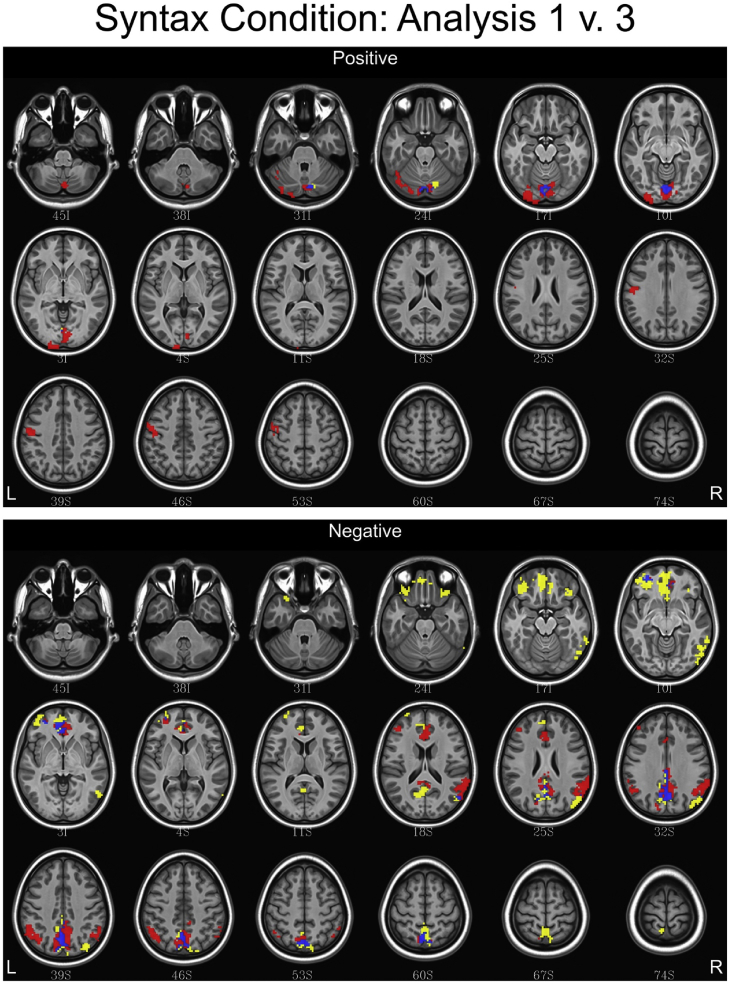
Conjunction maps showing the location of significant voxels positively or negatively associated with Analysis 1 and Analysis 3 for the syntax condition. Voxels associated with Analysis 1 are red. Voxels associated with Analysis 3 are yellow. Voxels common to both analyses are blue.

**Fig. 7:**
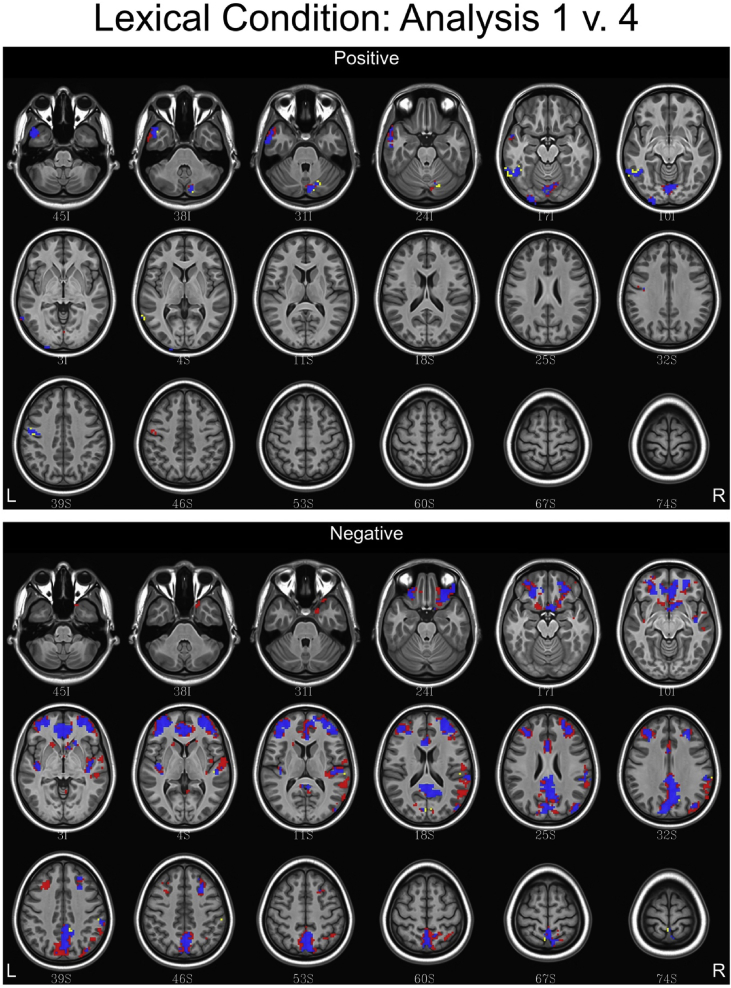
Conjunction maps showing the location of significant voxels positively or negatively associated with Analysis 1 and Analysis 4 for the lexical condition. Voxels associated with Analysis 1 are red. Voxels associated with Analysis 4 are yellow. Voxels common to both analyses are blue.

**Fig. 8:**
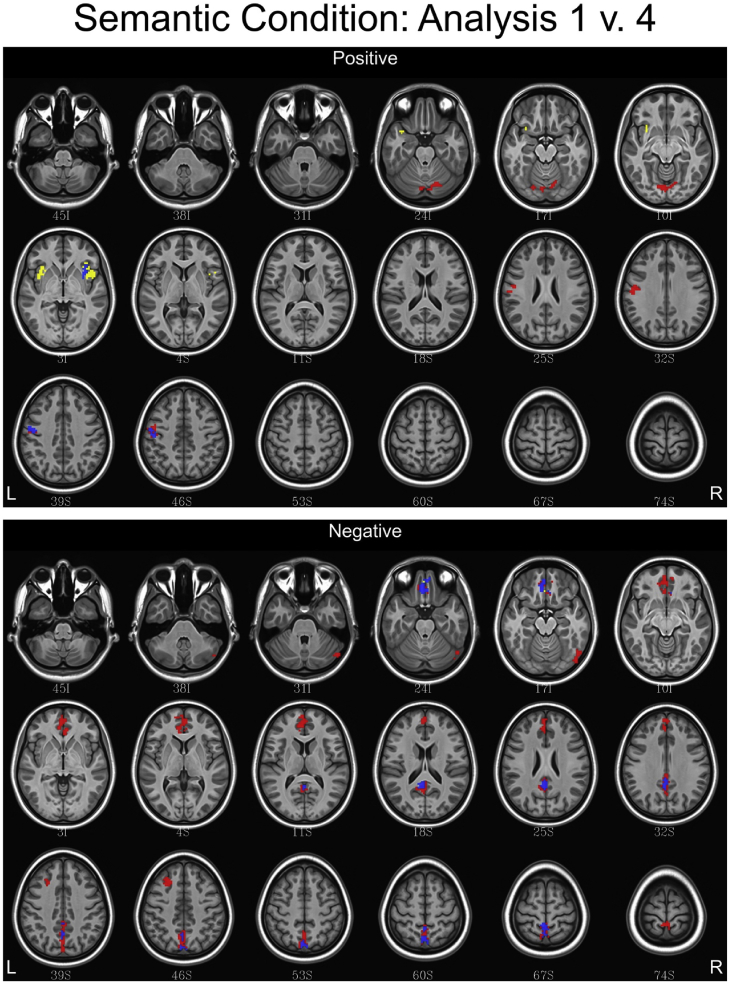
Conjunction maps showing the location of significant voxels positively or negatively associated with Analysis 1 and Analysis 4 for the semantic condition. Voxels associated with Analysis 1 are red. Voxels associated with Analysis 4 are yellow. Voxels common to both analyses are blue.

**Fig. 9:**
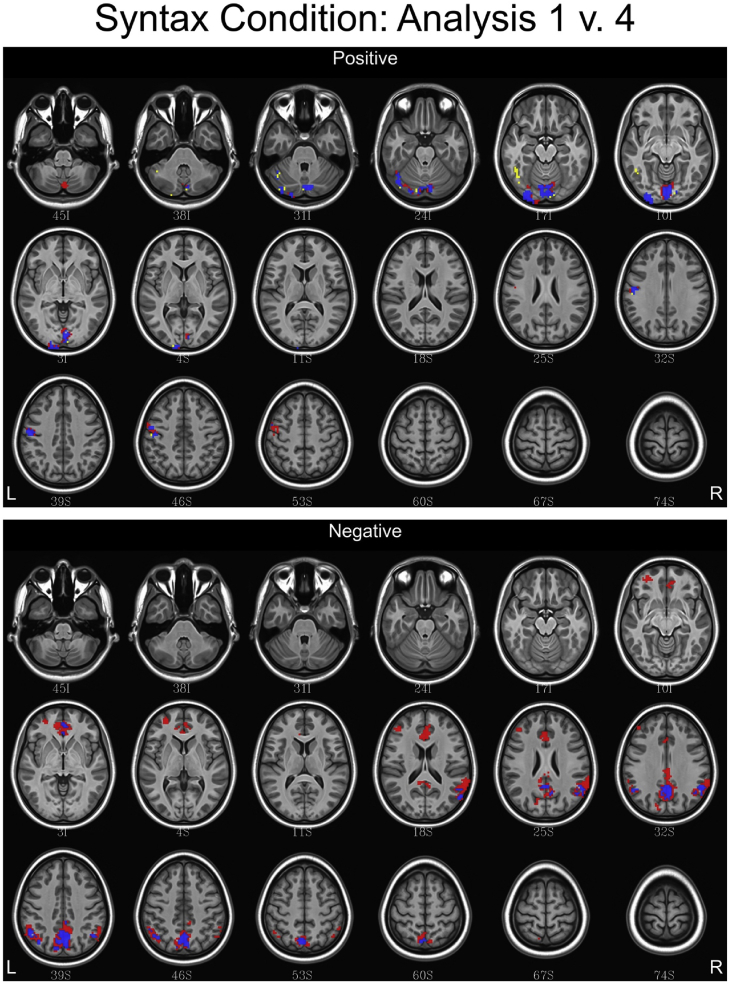
Conjunction maps showing the location of significant voxels positively or negatively associated with Analysis 1 and Analysis 4 for the syntax condition. Voxels associated with Analysis 1 are red. Voxels associated with Analysis 4 are yellow. Voxels common to both analyses are blue.

**Fig. 10:**
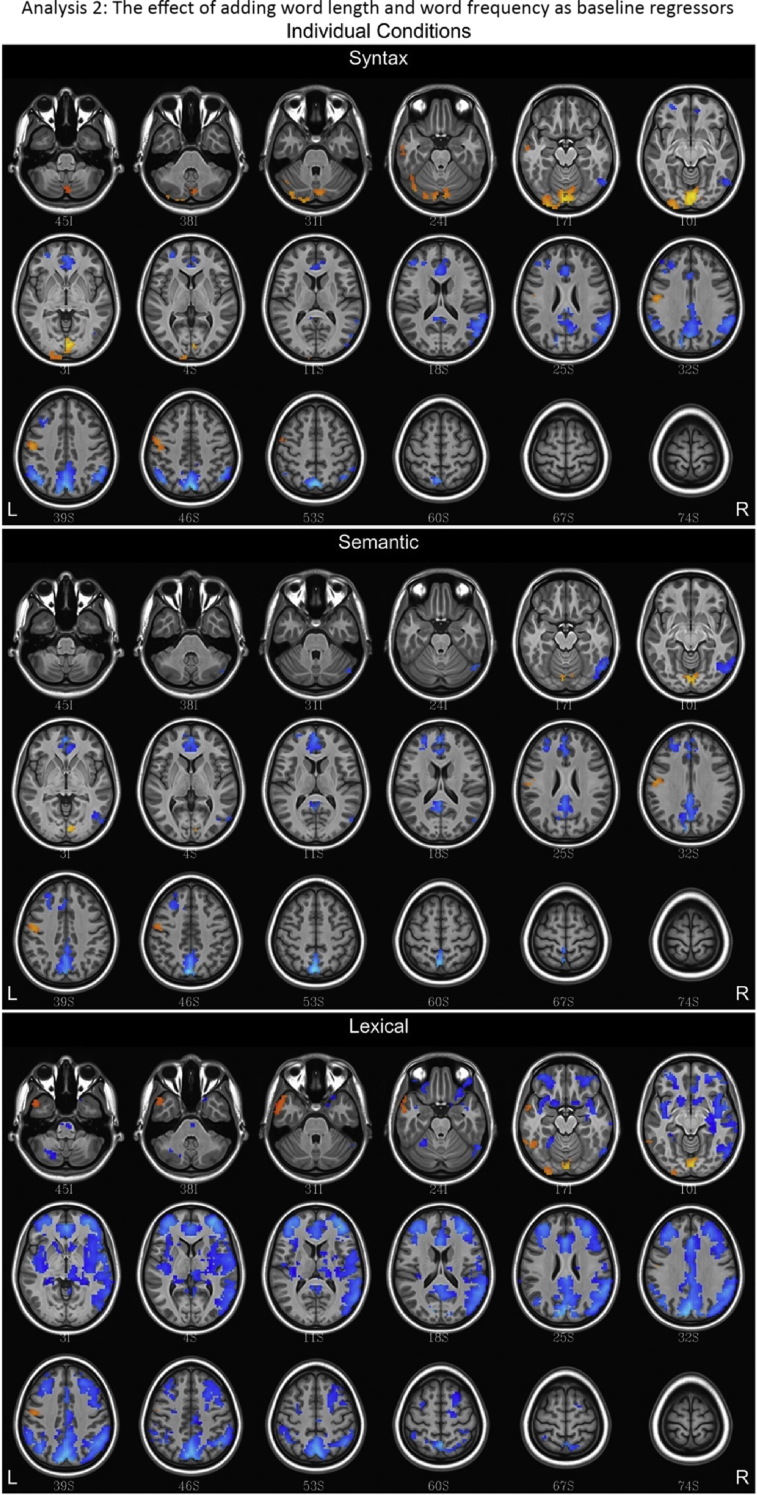
Fluctuations in the BOLD (blood oxygen level dependent) response for lexical, semantic and syntax conditions for Analysis 2, which incorporated word frequency and length into the baseline. Regions with a positive association are depicted in red/orange/yellow while those with a negative association are given a blue hue.

**Fig. 11:**
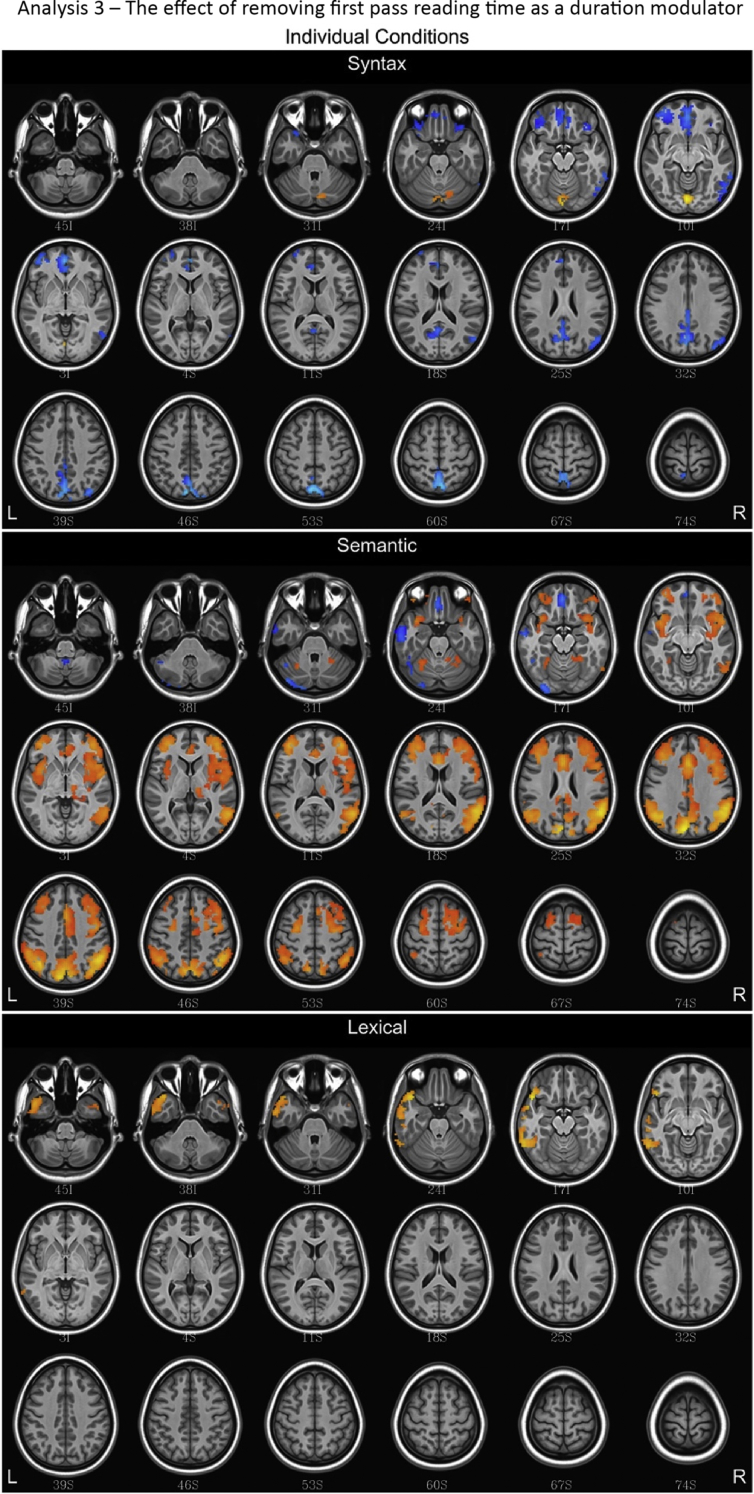
Fluctuations in the BOLD response for lexical, semantic and syntax conditions for Analysis 3, which omitted first pass reading time as an amplitude modulator. Regions with a positive association are depicted in red/orange/yellow while those with a negative association are given a blue hue.

**Fig. 12:**
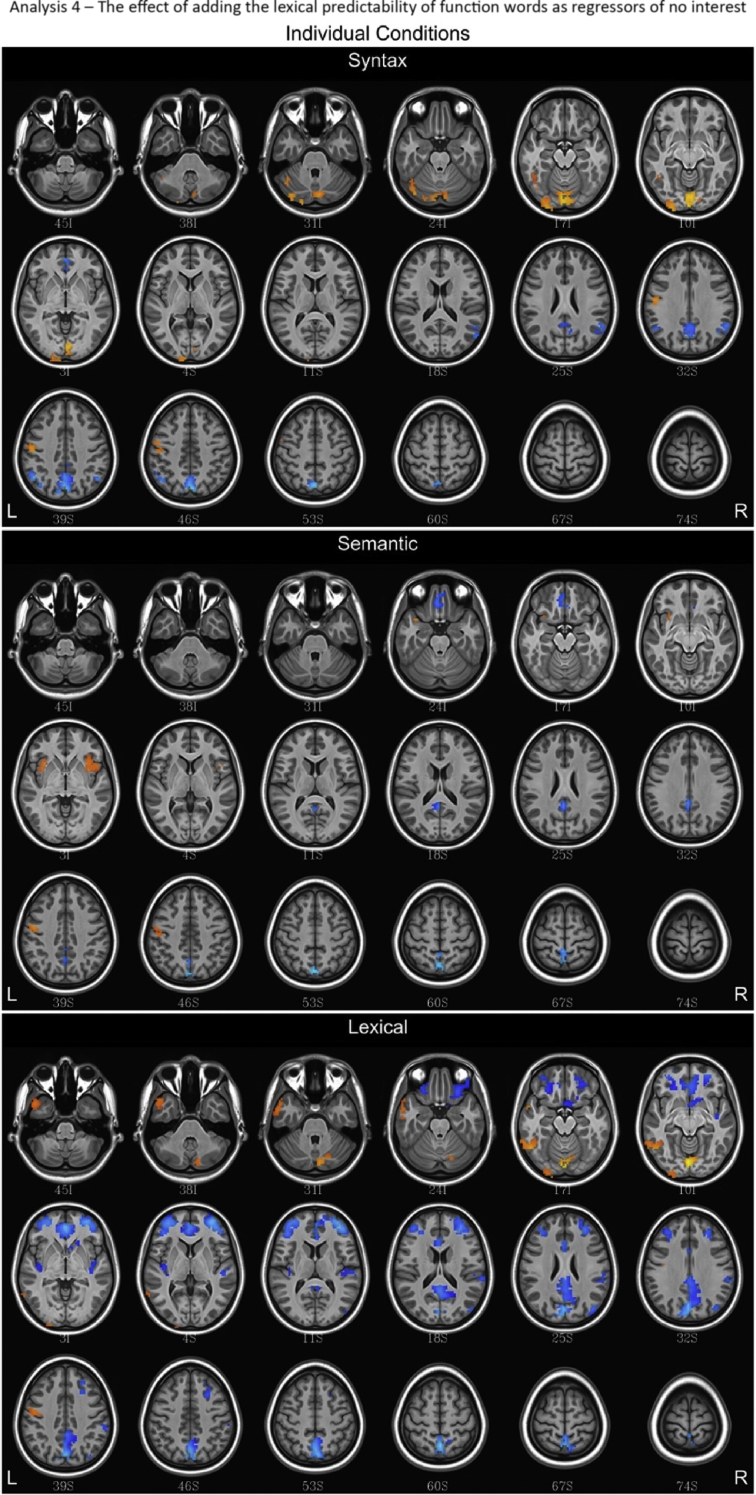
Fluctuations in the BOLD response for lexical, semantic and syntax conditions for Analysis 4, which included the lexical predictability of function words into the baseline. Regions with a positive association are depicted in red/orange/yellow while those with a negative association are given a blue hue.

Table 1 depicts the linguistic and eye tracking regressors used in each analysis. Fig. 1, Fig. 2, Fig. 3 contain conjunction maps created to compare the effect of convolving word length and frequency as baseline regressors to the primary dataset (found in Ref. [1]). Word length and frequency were added as baseline regressors because there is some evidence that these features such as word length have an independent influence on the oculomotor profile [6]. Incorporating these values into the secondary dataset produced statistical maps similar to the primary dataset, with a few differences noted in the semantic and syntax conditions. Therefore, incorporating word frequency and length into a baseline function may be of little utility. Fig. 4, Fig. 5, Fig. 6 contain conjunction maps demonstrating the effect of removing first pass reading time as a duration modulator from the primary dataset. Removing first pass reading time created a loosely fitted hemodynamic response function relative to the primary analysis and resulted in distinctly different statistical maps for all conditions. The most dramatic difference can be seen in the semantic condition in which the default mode network is now highly associated with this hemodynamic response function. This demonstrates the necessity of a tightly fitted hemodynamic response function that includes a duration modulation when using the oculomotor profile to study reading. Fig. 7, Fig. 8, Fig. 9 demonstrate the effect of including the lexical predictability of function words into the baseline function to the primary dataset. The inclusion of function words into the baseline response function is theoretically interesting as they are often skipped by the reader [7]. Including fixations on function words into the baseline response function resulted in statistical maps comparable to the primary dataset in both the lexical and syntax conditions. There were however differences in the semantic condition with the right and left anterior insula being associated. This deserves deeper investigation. Overall, this dataset demonstrates that focusing the analysis on content words is the best approach. Fig. 10, Fig. 11, Fig. 12 depict statistical maps of functional activity for each dataset that was compared to the primary analysis. Tables containing descriptive statistics for functional regions of interest in each dataset follow (see Table 2, Table 3, Table 4, Table 5, Table 6, Table 7, Table 8, Table 9, Table 10). These include volumetric data (how large activated regions of the brain were in microliters), max *z*-scores (the magnitude and direction of association with the hemodynamic profiles), MNI coordinates for the maximal intensities within each region (to allow for comparison with other data), anatomical and functional designations.

**Table 1:** Linguistic and eye tracking regressors utilized in each analysis.

Regressors of interest	Analysis 1	Analysis 2	Analysis 3	Analysis 4
Syntactic predictability	✓	✓	✓	✓
Semantic predictability	✓	✓	✓	✓
Lexical predictability	✓	✓	✓	✓
First pass reading time	✓	✓		✓
Regressors added to baseline				
Word length		✓		
Word frequency		✓		
Lexical predictability of function words				✓

Note: The results from Analysis 1 are contained in Carter et al. (2019, in press).

**Table 2:** Syntactic predictability in analysis 2.

Volume	Max *z*	MNI			Brodmann Area	Anatomical Structure
		x	y	z		
25785	−1.2332	1.5	−76.5	43.5	7	R. precuneus
18360	1.1921	1.5	−85.5	−7.5	18	R. lingual gyrus
17874	−0.8387	55.5	−49.5	43.5	39	R. inferior parietal lobe
9045	−0.6475	1.5	46.5	−1.5	10	R. anterior cingulate gyrus
6912	−0.8696	−46.5	−61.5	40.5	39	L. inferior parietal lobe
3456	0.769	−52.5	−13.5	37.5	4	L. precentral gyrus
3186	−0.5581	−37.5	28.5	28.5	9	L. middle frontal gyrus
2106	−0.4868	61.5	−64.5	−16.5	37	R. fusiform gyrus
1998	0.7165	−37.5	−82.5	−34.5		L. cerebellum
1755	−0.513	−43.5	40.5	22.5	10	L. middle frontal gyrus
1512	0.4411	−61.5	−13.5	−16.5	21	L. middle temporal gyrus
1080	−0.5511	−28.5	52.5	−4.5	10	L. superior frontal gyrus
1080	−0.6321	40.5	−79.5	31.5	19	R. inferior parietal lobe

Note: Locations of peak activation for each cluster with significant activity. The volume of each cluster (μl), peak z-score, MNI coordinates and anatomical and Brodmann's classifications are shown. L = left hemisphere, R = right hemisphere.

**Table 3:** Semantic predictability in analysis 2.

Volume	Max z	MNI			Brodmann Area	Anatomical structure
		x	y	z		
25677	−1.3036	−1.5	−76.5	49.5	7	L. precuneus
13203	−0.8868	−1.5	52.5	−1.5	32	L. anterior cingulate gyrus
8532	−0.6344	55.5	−64.5	−19.5	37	R. fusiform gyrus
6372	−0.5586	−25.5	52.5	16.5	10	L. superior frontal sulcus
2565	0.9323	−1.5	−85.5	−10.5	18	L. lingual gyrus
2538	0.7269	−49.5	−10.5	40.5	4	L. precentral gyrus

Note: Locations of peak activation for each cluster with significant activity. The volume of each cluster (μl), peak z-score, MNI coordinates and anatomical and Brodmann's classifications are shown. L = left hemisphere, R = right hemisphere.

**Table 4:** Lexical predictability in Analysis 2.

Volume	Max z	MNI			Anatomical Structure	Brodmann Area
		x	y	z		
265950	−1.1498	−7.5	−85.5	37.5	19	L. cuneus
36288	−0.7841	−31.5	49.5	28.5	10	L. middle frontal gyrus
16389	−0.4824	−46.5	1.5	−7.5	22	L. superior temporal gyrus
13419	−0.7142	−40.5	−67.5	37.5	39	L. inferior parietal lobe
5184	0.4668	−55.5	4.5	−16.5	38	L. superior temporal gyrus
3132	−0.3277	−16.5	4.5	−16.5	11	L. orbitofrontal cortex
2538	−0.2485	−16.5	−37.5	1.5	30	L. posterior cingulate gyrus
1890	0.9402	1.5	−85.5	−10.5	18	L. lingual gyrus
1863	−0.2847	−31.5	−61.5	−40.5		L. cerebellum
1728	−0.3605	−22.5	−49.5	−16.5	37	L. fusiform gyrus
1269	0.5993	−25.5	−100.5	−16.5	18	L. occipital lobe
1215	−0.3852	−28.5	−1.5	58.5	6	L. middle frontal gyrus
1134	−0.1838	−31.5	−40.5	−52.5		L. cerebellum
1080	−0.2971	4.5	−22.5	−49.5		R. medulla
1053	0.4592	−52.5	−58.5	−13.5	37	L. fusiform gyrus
1026	0.5672	−52.5	−13.5	34.5	4	L. precentral gyrus

Note: Locations of peak activation for each cluster with significant activity. The volume of each cluster (μl), peak z-score, MNI coordinates and anatomical and Brodmann's classifications are shown. L = left hemisphere, R = right hemisphere.

**Table 5:** Syntactic predictability in analysis 3.

Volume	Max *z*	MNI			Brodmann Area	Anatomical Structure
		x	y	z		
21330	−0.1526	−4.5	−70.5	55.5	7	L. precuneus
13068	−0.1256	−1.5	49.5	−1.5	32	L. anterior cingulate gyrus
9126	−0.0832	−40.5	52.5	−1.5	10	L. middle frontal gyrus
5076	0.1526	−1.5	−82.5	−10.5	18	L. lingual gyrus
4347	−0.087	40.5	−76.5	40.5	39	R. parietal lobe
3240	−0.0672	58.5	−64.5	−19.5	37	R. fusiform gyrus
1674	−0.052	34.5	28.5	−22.5	47	R. inferior frontal gyrus
1134	−0.0647	64.5	−49.5	−16.5	37	R. fusiform gyrus

Note: Locations of peak activation for each cluster with significant activity. The volume of each cluster (μl), peak z-score, MNI coordinates and anatomical and Brodmann's classifications are shown. L = left hemisphere, R = right hemisphere.

**Table 6:** Semantic predictability in analysis 3.

Volume	Max z	MNI			Brodmann Area	Anatomical structure
		x	y	z		
120609	0.3061	1.5	16.5	34.5	32	R. cingulate gyrus
52137	0.3646	58.5	−58.5	25.5	39	R. angular gyrus
30105	0.2619	−40.5	46.5	13.5	46	L. middle frontal gyrus
29592	0.4118	−7.5	−85.5	37.5	19	L. cuneus
28350	0.3607	−43.5	−61.5	34.5	39	L. angular gyrus
12393	0.2506	−43.5	7.5	−7.5	13	L. insula
7560	0.2246	−25.5	−7.5	52.5	6	L. superior frontal sulcus
3726	−0.1968	−61.5	−13.5	−19.5	21	L. middle temporal gyrus
3564	−0.2233	−31.5	−91.5	−16.5	18	L. occipital lobe
3267	−0.2362	−1.5	43.5	−19.5	11	L. superior frontal gyrus
2565	0.1484	10.5	−19.5	1.5	50	R. thalamus
2403	0.1266	−25.5	−55.5	−19.5		L. cerebellum
2133	0.1092	28.5	−49.5	−19.5	37	R. fusiform gyrus
1566	−0.164	−46.5	−49.5	−16.5	37	L. fusiform gyrus
1026	−0.2527	1.5	−46.5	−52.5		R. cerebellum

Note: Locations of peak activation for each cluster with significant activity. The volume of each cluster (μl), peak z-score, MNI coordinates and anatomical and Brodmann's classifications are shown. L = left hemisphere, R = right hemisphere.

**Table 7:** Lexical predictability in analysis 3.

Volume	Max z	MNI			Brodmann Area	Anatomical structure
		x	y	z		
13959	0.132	−49.5	19.5	−13.5	38	L. superior temporal gyrus
5670	0.1002	−61.5	−49.5	−16.5	37	L. fusiform gyrus
1053	0.0512	43.5	10.5	−40.5	38	R. middle temporal gyrus

Note: Locations of peak activation for each cluster with significant activity. The volume of each cluster (μl), peak z-score, MNI coordinates and anatomical and Brodmann's classifications are shown. L = left hemisphere, R = right hemisphere.

**Table 8:** Syntactic predictability in analysis 4.

Volume	Max z	MNI			Brodmann Area	Anatomical Structure
		x	y	z		
10881	−1.0339	1.5	−76.5	43.5	7	R. precuneus
8424	0.8619	−1.5	−82.5	−13.5	18	L. lingual gyrus
4374	0.6988	−25.5	−103.5	−13.5	18	L. occipital lobe
3267	0.6281	−40.5	−79.5	−25.5		L. cerebellum
3240	−0.6409	49.5	−61.5	40.5	39	R. inferior parietal lobule
2754	−0.6878	−49.5	−61.5	40.5	39	L. inferior parietal lobule
2403	0.576	−52.5	−13.5	37.5	4	L. precentral gyrus
1026	−0.6102	−1.5	49.5	−1.5	32	L. anterior cingulate gyrus
	−1.0339	1.5	−76.5	43.5	7	R. precuneus

Note: Locations of peak activation for each cluster with significant activity. The volume of each cluster (μl), peak z-score, MNI coordinates and anatomical and Brodmann's classifications are shown. L = left hemisphere, R = right hemisphere.

**Table 9:** Semantic predictability in analysis 4.

Volume	Max *z*	MNI			Brodmann Area	Anatomical structure
		x	y	z		
7452	−1.1302	−1.5	−76.5	52.5	7	L. precuneus
3267	−0.5464	−4.5	40.5	−19.5	11	L. orbitofrontal cortex
2700	0.4337	46.5	16.5	−4.5	13	R. anterior insula
2349	0.5394	−37.5	10.5	−22.5	38	L. superior temporal gyrus
1296	0.5245	−49.5	−7.5	40.5	6	L. posterior middle frontal gyrus

Note: Locations of peak activation for each cluster with significant activity. The volume of each cluster (μl), peak z-score, MNI coordinates and anatomical and Brodmann's classifications are shown. L = left hemisphere, R = right hemisphere.

**Table 10:** Lexical predictability in analysis 4.

Volume	Max *z*	MNI			Brodmann Area	Anatomical structure
		x	y	z		
32481	−0.8849	1.5	−58.5	61.5	7	R. precuneus
21114	−0.7026	31.5	55.5	1.5	10	R. middle frontal gyrus
15984	−0.5682	−37.5	52.5	4.5	10	L. middle frontal gyrus
13392	−0.8165	1.5	43.5	−4.5	32	R. anterior cingulate gyrus
4887	0.388	−55.5	4.5	−16.5	38	L. superior temporal gyrus
3483	0.8499	−1.5	−82.5	−10.5	18	L. lingual gyrus
3456	0.5542	−52.5	−58.5	−13.5	37	L. fusiform gyrus
2997	−0.3745	−1.5	4.5	−7.5	11	L. gyrus rectus
2160	−0.354	67.5	−31.5	22.5	40	R. superior temporal gyrus
2079	−0.437	43.5	−79.5	25.5	19	R. occipital lobe
1917	0.4193	−25.5	−103.5	−13.5	18	L. occipital lobe
1917	−0.3913	61.5	−34.5	31.5	40	R. supramarginal gyrus
1647	−0.2901	40.5	−16.5	−4.5	13	R. posterior insula
1512	−0.2819	−40.5	−16.5	4.5	13	L. anterior insula
1269	0.541	7.5	−82.5	−28.5		R. cerebellum
1161	0.3498	−49.5	−7.5	37.5	4	L. precentral gyrus

Note: Locations of peak activation for each cluster with significant activity. The volume of each cluster (μl), peak z-score, MNI coordinates and anatomical and Brodmann's classifications are shown. L = left hemisphere, R = right hemisphere.

Additional data can be accessed via GitHub (https://github.com/btcarter/LinguisticPrediction). Sample data from nine study participants are provided for the purpose of testing the scripts. This includes DICOM files from one structural image and three functional images per participant. Group statistical maps and conjunction maps for each dataset are also provided. The complete dataset can be found on the Open Science Framework (*osf.io/7csxr*).

## 2 Experimental design, materials and methods

### 2.1 Participants

Forty-three participants were recruited from the student body at Brigham Young University. All were right-handed, literate and native English speakers with 20/20 uncorrected or corrected vision without a history of reading disorders. Two participants were excluded due to eye tracking problems or excess motion in the scanner, resulting in a total of 41 participants included in the final analysis. Informed consent was obtained from all individuals prior to participation. The study was approved by the Brigham Young University Institutional Review Board ethics committee to ensure it conformed with the recognized ethical standards of the Declaration of Helsinki [8].

### 2.2 Materials

54 paragraphs were presented to participants during three functional scans (18 paragraphs per scan). These paragraphs were a subset of those created for the Provo Corpus [9] and their linguistic predictability characteristics were previously characterized via cloze procedure [9], [10], [11] and latent semantic analysis [12]. Linguistic predictability refers to the probability that a word may be accurately predicted given the preceding text and can be computed in terms of lexical (whole word form), semantic (word meaning), syntactic (word class) values.

The cloze procedure is a simple method of computing how expected a word is given its preceding context or predictability. Participants are presented with the first word of a sentence and asked what the following word will be. Their response is recorded and then the word is revealed. At this point they are asked what the third word in the sentence will be, and so on until responses have been gathered for each word in the text. Responses are then scored according to whether they match the word class (syntax), and whole word form (lexical) of the target word. The fraction of correct responses for each characteristic results in a predictability score for that characteristic. E.g. if participants were asked what word might follow the phrase “I want to drive the” and 50% responded “car”, 30% responded “truck”, 15% responded “train” and 5% “forklift” (the correct response was “car”) then this word would be scored as having a lexical predictability of 0.5 (only 50% of respondents answered “car”) and syntactic predictability of 1.0 (all respondents answered with a noun).

### 2.3 Apparatus

Paragraphs were presented to participants via Cambridge Systems MRI-safe LCD monitor located at the end of the scanner bore and viewed via a mirror attached to the head coil. Screen resolution was set for 1600x1200. Text was displayed in Courier New font at 26pt, resulting in approximately 4 letters per degree of visual angle. Eye-movements were recorded via an SR Research Eyelink 1000 plus long-range MRI eye tracker sampling at 1000 Hz (Eyelink 1000, SR research, Mississauga, Canada). A Siemens 3T Tim Trio with a 12-channel receive only head coil was used for this study. Software version was syngo MR B17.

### 2.4 Procedure

#### 2.4.1 Eye-movement data acquisition

Only movements of the right eye were recorded, though viewing was binocular. Prior to the beginning of each scan, participants completed a nine-point calibration and validation exercise. An average error of 0.49° and a maximum error of 0.99° of visual angle were required to pass. A single trial consisted of viewing a fixation cross for 6 seconds, followed by a paragraph, which was viewed for 12 seconds. Stimulus presentation and eye position were controlled and recorded via SR. Research software. Eye movements were co-registered with scanner sequence. The experiment was programmed to begin once an onset signal had been received from the scanner control computer. All fixation times were computed relative to this signal.

#### 2.4.2 Scan Sequence

The following scans were performed, listed in order: a localizer, 3 consecutive 5.66 minute functional scans, followed by a structural scan.

*Functional scan parameters.* Interleaved, T_2_*-weighted echo-planar imaging protocol: slice number = 43, transverse orientation, phase encoding was anterior to posterior with 0° rotation, FOV = 224 × 224mm, acquisition matrix = 64x64, slice thickness = 3.00mm, TR = 2500 ms, and TE = 28 ms, 134 repetitions with a flip angle of 9°.

*Structural scan parameters.* a T_1_-weighted, magnetization prepared rapid gradient-echo (MPRAGE) protocol: orientation = sagittal, anterior to posterior phase encoding, FOV = 218×250, matrix = 256x256, slice thickness = 1mm, TR = 1900 ms, TE = 2.26 ms and flip angle = 9°.

### 2.5 Analysis

#### 2.5.1 fMRI

Analysis of Functional NeuroImages (AFNI) [13], version 17.0.16 and Advanced Normalization Tools (ANTs) [14] were used. DICOM images were converted to BRIK and HEADER files via to3d. The structural scan was then co-registered to the third functional scan via 3dWarp. 3dTshift was used for slice time correction. Functional scans were corrected for low-frequency motion by aligning all volumes to the middle acquisition volume. Blocks were aligned to the same functional space via 3dvolreg. A skullstripped mask was created for each subject using 3dSkullStrip and used to restrict the analysis to only brain matter. Input matrices were constructed and decoded via 3dDeconvolve. Each analysis had 6 polynomial regressors for motion. This included pitch, roll, yaw, superior-inferior translation, left-right translation, and anterior-posterior translation. Additional regressors were added for each dataset. Timing series coding these regressors were constructed from the eye tracking data via R [15], version 3.3.2.

*Analysis 1*. Three parametric regressors were added encoding lexical predictability, semantic predictability, and syntactic predictability. First pass reading time (the amount of time spent with the fovea fixed upon a word when the word is first encountered) was used as a duration modulator for each regressor. Log transformations were applied to lexical and syntactic predictability measures. Semantic predictability was not log transformed (see Ref. [1] for an explanation).

*Analysis 2.* This included all the regressors used for Analysis 1 with additional regressors coding for word length and word frequency. These regressors were added to baseline as amplitude modulated hemodynamic response functions. Each regressor was fitted using fixation onset to mark the beginning of each event with word length or frequency acting as the amplitude of the function (word frequency was log transformed).

*Analysis 3.* This omitted first pass reading time as a duration modulator. All other regressors were the same as that found in Analysis 1.

*Analysis 4.* This incorporated the lexical predictability of function words into the baseline hemodynamic response function, in addition to the regressors incorporated in Analysis 1.

Deconvolution was performed via 3dDeconvolve. A 5mm blur was applied to the output via 3dmerge and individual anatomical and statistical maps were projected into MNI_ICBM152 space [16], [17] via ants.sh [14]. A binary map group map was then constructed and used to exclude white matter. 3dttest++ was used to apply a random effects analysis and compute cluster thresholds via the option “-Clustsim”. A voxel-wise threshold of *p <* 0.001 and a cluster-threshold of 38 voxels were used to achieve an α < 0.05 [18]. 3dclust was used to compute descriptive statistics and coordinates of peak activity.

#### 2.5.2 Conjunction map construction

Masks were created from the statistical maps created during the random effects analysis, and overlaid via 3dcalc [13] to visualize cluster overlay. Regions pertaining to the first analysis were given a value of 1, those pertaining only to the second the value of 2. This resulted in common regions being given a value of 3. A t-statistic threshold of 3.291 was used.

### 2.6 Scripts

All analysis scripts are available at: https://github.com/btcarter/LinguisticPrediction/. Additional information concerning script implementation, execution, and sample data, can be found here.
